# RF-PCA: A New Solution for Rapid Identification of Breast Cancer Categorical Data Based on Attribute Selection and Feature Extraction

**DOI:** 10.3389/fgene.2020.566057

**Published:** 2020-09-09

**Authors:** Kai Bian, Mengran Zhou, Feng Hu, Wenhao Lai

**Affiliations:** ^1^School of Electrical and Information Engineering, Anhui University of Science and Technology, Huainan, China; ^2^State Key Laboratory of Mining Response and Disaster Prevention and Control in Deep Coal Mines, Anhui University of Science and Technology, Huainan, China

**Keywords:** breast cancer, artificial intelligence, random forest, principal component analysis, extreme learning machine

## Abstract

Breast cancer is one of the most common cancer diseases in women. The rapid and accurate diagnosis of breast cancer is of great significance for the treatment of cancer. Artificial intelligence and machine learning algorithms are used to identify breast malignant tumors, which can effectively solve the problems of insufficient recognition accuracy and long time-consuming in traditional breast cancer diagnosis methods. To solve these problems, we proposed a method of attribute selection and feature extraction based on random forest (RF) combined with principal component analysis (PCA) for rapid and accurate diagnosis of breast cancer. Firstly, RF was used to reduce 30 attributes of breast cancer categorical data. According to the average importance of attributes and out of bag error, 21 relatively important attribute data were selected for feature extraction based on PCA. The seven features extracted from PCA were used to establish an extreme learning machine (ELM) classification model with different activation functions. By comparing the classification accuracy and training time of these different models, the activation function of the hidden layer was determined as the sigmoid function. When the number of neurons in the hidden layer was 27, the accuracy of the test set was 98.75%, the accuracy of the training set was 99.06%, and the training time was only 0.0022 s. Finally, in order to verify the superiority of this method in breast cancer diagnosis, we compared with the ELM model based on the original breast cancer data and other intelligent classification algorithm models. The algorithm used in this article has a faster recognition time and a higher recognition accuracy than other algorithms. We also used the breast cancer data of breast tissue reactance features to verify the reliability of this method, and ideal results were obtained. The experimental results show that RF-PCA combined with ELM can significantly reduce the time required for the diagnosis of breast cancer, which has the ability of rapid and accurate identification of breast cancer and provides a theoretical basis for the intelligent diagnosis of breast cancer.

## Introduction

Cancer is a disease that seriously threatens human health. The latest annual report on cancer incidence in the United States (Siegel et al., 2020) shows that it is estimated that in 2020, 1,806,590 new cancer cases will be found in the United States, which is equivalent to nearly 5,000 people suffering from cancer every day. There will be 606,520 cancer deaths, which is equivalent to more than 1,600 cancer deaths per day. Over the most recent 5−year period (2012–2016), the breast cancer incidence rate increased slightly by 0.3% per year (DeSantis et al., 2019). Cancer not only affects people’s normal life but also brings a huge economic burden to people with high medical costs. Therefore, more and more researchers are committed to the research of cancer diagnosis and treatment methods (Gebauer et al., 2018). Among them, the incidence rate of breast cancer is only second after the lung cancer incidence rate in the world (Wang et al., 2018). Early detection and diagnosis of breast cancer are very helpful for treatment. If breast cancer is detected early, it can guide clinically targeted prevention and treatment measures, reduce the recurrence rate of breast cancer, improve the prognosis of patients, and prolong the life cycle of patients (Charaghvandi et al., 2017). How to quickly and accurately predict breast malignant tumors has become the key to the breast cancer diagnosis.

The traditional diagnosis method of breast cancer is mainly a fine-needle aspiration cell method (Dennison et al., 2015). The degree of canceration can be determined by observing the abnormal cell morphology of the collected tissue sections under the light microscope. This method needs the operation of experts with senior clinical experience, but it may cause the wrong diagnosis due to various uncertain subjective factors, which will also consume a lot of working time. In recent years, various prediction algorithms in machine learning can be well used in disease diagnosis, and more intelligent prediction results can be used to assist doctors, so as to speed up the time of diagnosis and improve the accuracy of diagnosis. For example, Cui et al. (2018) used neural network cascade (NNC) model identified numerous candidate miRNA biomarkers to detect breast cancer and obtained equivalent diagnostic performance. Wang et al. (2017) used a support vector machine (SVM)-based weighted AUC ensemble learning model to achieve a reliable and robust diagnosis of breast cancer. Noorul et al. (2019) proposed a transfer learning-based deep convolutional neural network (CNN) for segmentation to improve the detection rate of breast cancer for histopathological images. However, most of these machine learning algorithms analyze all the attributes of breast cancer data, which fails to take into account the influence of redundant information on the experimental results and the relationship between the attribute factors. Deep learning algorithm used to detect breast cancer needs to analyze the histopathological images of breast cancer, which not only requires a large number of samples, but also consumes a lot of time, and the prediction efficiency is low. Some artificial intelligence algorithms and classification models have been proposed to identify breast malignant tumor by using the Wisconsin Breast Cancer Database (WBCD). For example, Sewak et al. (2007) provide a resemble learning method based on SVMs to classify the breast malignant tumor and achieved with acceptable prediction accuracy. Nahato et al. (2015) combined rough set indiscernibility relation method with back propagation (BP) neural network for analysis of breast cancer dataset and the breast cancer dataset obtained its higher performance with a reduct of least number of attributes. Mert et al. (2014) used the independent component analysis and the discrete wavelet transform to reduce the dimension of data. A probabilistic neural network (PNN) classification model is established to increases the performance of breast cancer classification as benign and malignant and reduce the computational complexity. Jhajharia et al. (2016) used the principal component analysis (PCA) to preprocess the original breast cancer data, and then a decision tree (DT) prediction model was established to achieve the prognostic analysis of breast cancer data. Yang and Xu (2019) developed a feature extraction method by PCA and a differential evolution algorithm to optimize the parameter of SVM for the identification of breast tumors to present a superior classification performance.

Random forest (RF) is a supervised learning algorithm, which can select features according to the importance of attributes and reduce the complexity of the model (Odindi et al., 2014). Saraswat and Arya (2014) introduced a novel Gini importance-based binary random RF selection method to extract the relevant features of leukocytes and got a high classification accuracy. Zhou et al. (2017) proposed an iterative RF method to select candidate biomarkers and completed the classification of renal fibrosis. Wade et al. (2016) used the regularized RF to select the features of high dimensional shape data from subcortical brain surfaces. PCA is a kind of unsupervised learning feature extraction algorithm which maps high-dimensional data to low-dimensional space by linear projection and reduces the dimension of data sets (Simas Filho and Seixas, 2016). Skala et al. (2007) chose a method based on PCA to use the information inherent in the dose-volume histograms (DVH) to analyze after image-guided radiation therapy for prostate cancer. Fabris et al. (2014) employed sparse PCA to assess the glucose variability index of continuous glucose monitoring (CGM) time-series. Garbis et al. (2018) used PCA for proteomic quantitative analysis of primary cancer-associated fibroblasts in esophageal adenocarcinoma. Extreme learning machine (ELM) is an efficient and intelligent algorithm that can be used to solve classification or regression problems (Huang et al., 2010). Kavitha et al. (2015) combined the ELM with fractal feature analysis to assess glaucoma. Bueno-Crespo et al. (2017) put forward a method based on ELM to automatically design a multitask learning machine. At present, most of the researches are to use feature selection and feature extraction methods independently, but we combine feature extraction and feature selection to carry out the follow-up research work in this article.

The present work is concerned with the development of analytical method for rapid identification of breast cancer categorical data based on attribute selection and feature extraction. Firstly, the RF is used for characteristic attribute selection processing of original breast cancer data, and the samples are divided into a training set and test set. Then, feature extraction and dimensionality reduction of selected attribute data by the PCA. Finally, the extracted characteristic data are used as the input of the ELM to establish the identification model of breast malignant tumor. Brief conclusions and future work are summarized at the end of the article.

## Materials and Methods

### Collection of Breast Cancer Data

The validity and feasibility of the methods described in this article were verified by the University of Wisconsin breast cancer data sets (Street et al., 1993). There are 569 cases of breast tumor data in this nuclear micrograph of breast tumor lesion tissue database, including 357 cases of benign tumors and 212 cases of malignant tumors. To facilitate the proportional division of samples, 400 cases were randomly selected as the study objects, including 200 cases of benign tumors and 200 cases of malignant tumors. The quantitative real-valued features of the nuclear micrograph of breast tumor lesion tissue include radius (mean of distances from center to points on the perimeter), texture (standard deviation of gray-scale values), perimeter (sum of the distances between consecutive boundary points), area (perimeter to compensate for digitization error), smoothness (local variation in radius lengths), compactness (perimeter^2^/area − 1.0), concavity (severity of concave portions of the contour), concave points (number of concave portions of the contour), symmetry (relative difference in length between pairs of line segments perpendicular to the major axis), and fractal dimension (“coastline approximation” − 1). A set of data for each case includes 30 attributes, including the average value, standard deviation, and worst value (the average value of the three largest data of each feature) of the 10 characteristic quantities of each nucleus in the sampled tissue. The 30 attributes were already present from the data sets. Each sample data is composed of 32 fields. The first field is case number, the second field is diagnosis result, B is benign, M is malignant. The other fields are all the attributes of 10 quantitative features, and the first to the tenth attributes are the average value of 10 quantitative features. The 11th to 20th attributes are the standard deviation of 10 quantitative features. The 21st to 30th attributes are the worst value (average value of the three largest data of each feature) of 10 quantitative features. These characteristics can reflect the nature of the breast tumor.

The hardware conditions of the computer used in the experiment include an Intel Core i7 processor, an NVIDIA RTX 2070 graphics card, and a 16G Kingston memory module, etc. The algorithm simulation is run in MATLAB R2016b (MathWorks, United States) environment.

### Random Forest for Attribute Selection

The feature selection method is to select features from the original attribute data and get a new feature subset composed of the original features, so as to reduce the number of attributes in the attribute set. It is an inclusive relationship and does not change the original feature space (Guyon and Elisseeff, 2003). RF is a supervised learning algorithm that uses multiple DT to train samples. This algorithm was proposed by Breiman (2001), which can be used to solve classification and regression problems. The RF feature selection method will give the importance score of each variable (Genuer et al., 2010), evaluate the role of each variable in the classification problem, and delete the attribute with lower importance. If a feature is randomly added with noise, the accuracy of out of bag data changes significantly, which shows that this feature has a greater impact on the predictive results of samples. Furthermore, it shows that its importance is high, so it is necessary to select and delete the attributes with low importance. The out of bag error (Mitchell, 2011) is usually used to evaluate the importance of features by RF.

The steps for attribute selection of RF algorithm are as follows:

*Step 1:* calculate the importance of each attribute and arrange it in descending order of importance

Attribute importance *I*_*m*_:

(1) $$I_{m}=\frac{1}{N}\,\sum \left(e\,r\,r\,O\,O\,B\,2-e\,r\,r\,O\,O\,B\,1\right)$$

Where, *N* is the tree in the RF, *errOOB2* represents the out of bag error of data with noise interference, and *errOOB1* denotes the out of bag error of original data;

*Step 2:* Set the threshold value, delete the attributes whose importance is lower than the threshold value from the current attributes, and the remaining attributes will form a new attribute set again;

*Step 3:* A new RF is established by using the new attribute set, the importance of each attribute in the attribute sets are calculated and arranged in descending order;

*Step 4:* Repeat *step 2* and *step 3* until all the attribute importance values are greater than the threshold value;

*Step 5:* Each attribute set corresponds to a RF, and the corresponding out of bag error rate is calculated;

*Step 6:* Take the attribute set with the lowest out of bag error rate as the last selected attribute set.

### Normalization of Data

Standardization refers to the pre-processing of data so that the values fall into a unified range of values. In the process of modeling, the difference of each feature amount is reduced (He et al., 2010). Different data often have different dimension units and do not belong to the same order of magnitude. The data with a too-large difference will eventually affect the evaluation results. To eliminate the influence of too large dimensional difference between indicators, before using PCA for feature selection, data need to be standardized to solve the error caused by the difference between data indicators (Sun et al., 2016). The common standardization methods are Min − Max normalization (Snelick et al., 2005) and *Z*-score normalization (Ribaric and Fratric, 2006). Min–max normalization can normalize data to interval [0, 1] and interval [−1, 1] respectively.

[0,1] normalization:

(2) $$X_{\left[0,1\right]}=\frac{X-X_{M\,i\,n}}{X_{M\,a\,x}-X_{M\,i\,n}}$$

[−1,1] normalization:

(3) $$X_{\left[-1,1\right]}=\frac{X-\mu }{X_{M\,a\,x}-X_{M\,i\,n}}$$

*Z*-score normalization:

(4) $$X_{Z}=\frac{X-\mu }{\sigma }$$

Where *X* is the original sample data, *X*_*Max*_ is the maximum value of the original sample data, *X*_*Min*_ is the minimum value of the original sample data, μ denotes the average value of the original sample data, and σ represents the standard deviation of the original sample data.

### Principal Component Analysis for Feature Extraction

The method of feature extraction is mainly to transform the feature space through the relationship between attributes, map the original feature space to the low-dimensional feature space, so as to complete the purpose of dimension reduction (Wang and Paliwal, 2003). As an unsupervised learning dimensionality reduction method, PCA reduces the data dimension through the correlation between multidimensional data groups. On the premise of minimizing the information loss, it can simplify the data structure, make the data set easier to use, completely without parameter limitation, and reduce the calculation cost of the algorithm (Hess and Hess, 2018).

The steps of the PCA algorithm for feature extraction are as follows:

*Step 1:* Input the original sample data matrix *X*:

(5) $$X=\left(\begin{array}{cccc} x_{11} & x_{12} & \cdots & x_{1\,n}\\ x_{21} & x_{22} & \cdots & x_{2\,n}\\ \vdots & \vdots & \vdots & \vdots \\ x_{m\,1} & x_{m\,2} & \cdots & x_{m\,n} \end{array}\right)$$

*Step 2:* Set each column as a feature and average each feature. Subtract the average value from the original data to the new centralized data;

*Step 3:* Calculate the covariance matrix:

(6) $$D\,\left(X\right)=\frac{1}{n}\,X\,X^{T}$$

*Step 4:* Solve eigenvalue λ and eigenvector *q* of covariance matrix by the eigenvalue decomposition method;

*Step 5:* Sort the eigenvalues from large to small, and select the largest *k* of them. Then the corresponding *k* eigenvectors are used as row vectors to form eigenvector matrix *Q*;

*Step 6:* Multiply the data set *m*^∗^*n* by the eigenvector of *n* dimensional eigenvector, and obtain the data matrix *Y=QX* of the last dimension reduction.

As the basis of selecting the number *k* of principal components, the cumulative contribution rate of principal components is generally required to be more than 85%.

### Extreme Learning Machine for Classification

**FIGURE 1:**
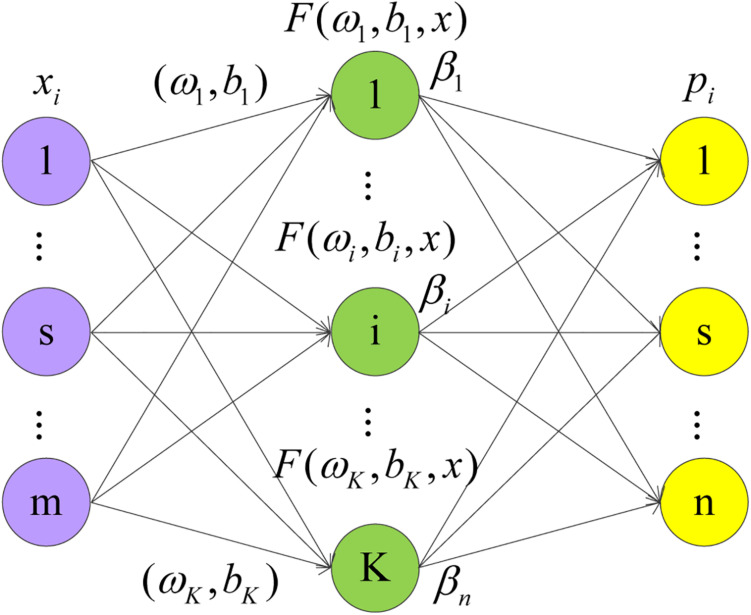
Network structure diagram of ELM.

The ELM is a simple and efficient learning algorithm proposed by professor Huang (Huang et al., 2006) of the Nanyang Polytechnic, it can be used to solve the problem of classification and regression in pattern recognition. This algorithm only needs to set the number of hidden layer neurons of the network, it does not need to adjust the input weight of the network and the bias of hidden layer neurons in the process of implementation, and produces a unique optimal solution, so the learning speed is fast and the generalization performance is good (Zhang and Ding, 2017). ELM is a single-layer feedforward network that can train training set quickly. There are only three layers in the network, namely the input layer, the hidden layer, and the output layer. The network structure of ELM is shown in Figure 1. From left to right, there are input layer neurons, hidden layer neurons, and output layer neurons.

There are *S* different training samples s, where *x*_*i*_ = [*x*_1_,*x*_2_,*x*_3_,⋯,*x*_*m*_]^*T*^, *x*_*i*_ ∈ *Rm*.*p*_*i*_ = [*p*_1_,*p*_2_,*p*_3_,⋯,*p*_*n*_], *t*_*i*_ ∈ *Rn*. Set the activation function *g*(*x*), with *K* hidden layer nodes output as follows:

(7) $$p_{i}=\sum_{i=1}^{K}\beta_{i}\,g\,\left(\omega_{i}\cdot x_{j}+b_{i}\right)=\sum_{i=1}^{K}\beta_{i}\,F\,\left(\omega_{i},b_{i},x_{j}\right)$$

Where *j* = 1,2,⋯,*N*, ω_*i*_ = [ω_1_,ω_2_,⋯,ω_*m*_]^*T*^ is the input weight of the hidden layer neuron, *b*_*i*_ is the hidden layer neuron bias, and β_*i*_ = [β_1_,β_2_,⋯,β_*n*_]^*T*^ is the output weight of the output neuron.

The steps of the ELM algorithm are as follows:

*Step 1:* Select (ω_*i*_,*b*_*i*_) randomly and map the samples to the feature space according to *h*(*x*) = [*F*(ω_1_,*b*_1_,*x*),⋯*F*(ω_*K*_,*b*_*K*_,*x*)]^*T*^. If the feature mapping *h*(*x*) forms the hidden layer matrix *H*, then it exists

(8) H⁢β=P

$$\text{Where }H\;=\;\left[\begin{array}{l} h(x_{1})\\ \vdots \\ h(x_{S}) \end{array}\right]\;=\;{\left[\begin{array}{l} \begin{array}{ccc} {\text{F(ω}}_{1}\text{, }b_{1}\text{, }x_{1}\text{)} & \cdots & {\text{F(ω}}_{K}\text{, }b_{K}\text{, }x_{1}\text{)} \end{array}\\ \begin{array}{ccc} {\text{F(ω}}_{1}\text{, }b_{1}\text{, }x_{S}\text{)} & \cdots & {\text{F(ω}}_{K}\text{, }b_{K}\text{, }x_{S}\text{)} \end{array} \end{array}\right]}_{S\times K},\text{ β = }{\left[\begin{array}{l} {\text{β}}_{1}^{\text{T}}\\ \vdots \\ {\text{β}}_{K}^{\text{T}} \end{array}\right]}_{K\times n}\text{ and }P\;=\;{\left[\begin{array}{l} p_{1}^{\text{T}}\\ \vdots \\ p_{S}^{\text{T}} \end{array}\right]}_{S\times n}.$$

Sin function, Hardlim function, and Sigmoid function can be selected as the activation function of hidden layer neurons (Song et al., 2015).^∗^

*Step 2:* In the new feature space, the optimal output weight β^∗^ is obtained from Eq. (8) by using the least square method, where *H*^+^ is the Moore-Penrose generalized inverse of *H*, β^∗^ = H^+^*P*.

### Evaluation Index of Classifier Performance

In order to better evaluate the performance of classifier, we introduce the confusion matrix. In the field of machine learning, confusion matrix is a visual tool to evaluate the performance of classification models. Among them, each column of the matrix represents the situation of predictive samples and each row of the matrix represents the situation of actual samples (Deng et al., 2016). The confusion matrix consists of true positive (TP), false positive (FP), true negative (TN), and false negative (FN). The accuracy, precision, sensitivity, specificity, F1-score and MCC (Azar and El-Said, 2012; Zheng et al., 2018) can be obtained from the confusion matrix and all of them are used as evaluation indexes of performance. In general,

Accuracy is the ratio of the correctly classified examples to the total sample size.

(9) $$A\,c\,c\,u\,r\,a\,c\,y=\frac{T\,P+T\,N}{T\,P+F\,N+F\,P+T\,N}$$

Precision is the percentage of samples are correctly classified as true positive.

(10) $$P\,r\,e\,c\,i\,s\,i\,o\,n=\frac{T\,P}{T\,P+F\,P}$$

Sensitivity is the percentage of samples are correctly classified as true positive in total positive samples.

(11) $$S\,e\,n\,s\,i\,t\,i\,v\,i\,t\,y=\frac{T\,P}{T\,P+F\,N}$$

Specificity is the percentage of samples are correctly classified as true negative in total negative samples.

(12) $$S\,p\,e\,c\,i\,f\,i\,c\,i\,t\,y=\frac{T\,N}{T\,N+F\,P}$$

F1-score is an index used to measure the accuracy of a binary classification model.

(13) $$F\,1-s\,c\,o\,r\,e=\frac{2*P\,r\,e\,c\,i\,s\,i\,o\,n*S\,e\,n\,s\,i\,t\,i\,v\,i\,t\,y}{T\,N+F\,P}$$

MCC is essentially a balanced index that describes the correlation coefficient between the actual classification and the predicted classification, which is used to measure the classification performance of binary classification. The value range of MCC is [−1,1]. The closer the MCC value is to 1, the better the classifier performance.

(14) $$M\,C\,C=\frac{T\,P\cdot T\,N-F\,P\cdot F\,N}{\sqrt{\left(T\,P+F\,P\right)\,\left(T\,P+F\,N\right)\,\left(T\,N+F\,P\right)\,\left(T\,N+F\,N\right)}}$$

## Results and Discussion

### Attribute Selection Based on Random Forest

There are 30 attributes in the original breast cancer data, each of which contains the corresponding information of breast tumor lesion tissue. Different attributes play different roles in the analysis of breast cancer data. Redundant and less important attributes will affect the establishment of breast cancer of a predictive model, which cannot achieve high prediction accuracy, but also increase the complexity of the model and reduce the efficiency of breast cancer prediction. Attribute selection based on RF of the method is used to select more important attributes to improve the efficiency of modeling and prediction ability. Before RF is used, we set the number of trees to 200, the number of leaf node samples to 1, and the number of fboot to 1.

**FIGURE 2:**
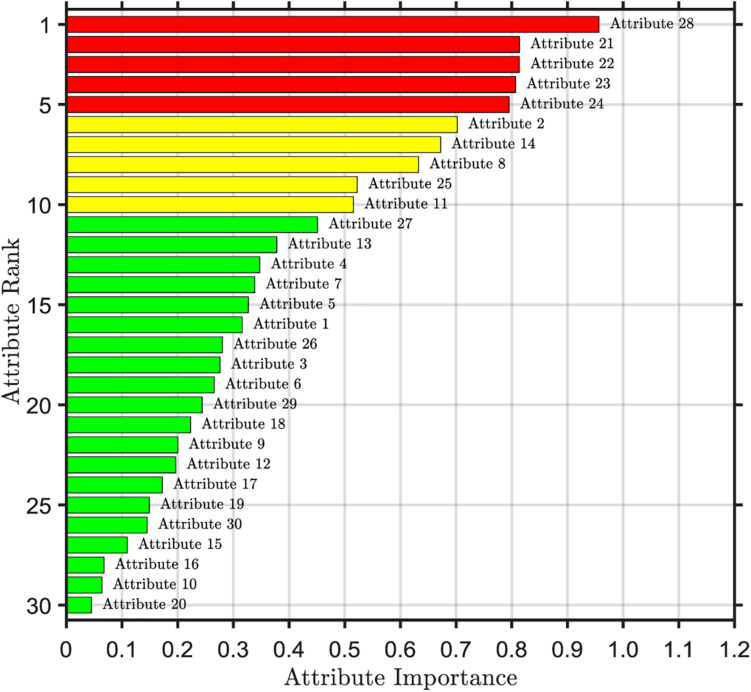
Ranking of attribute importance for RF initial selection.

The importance ranking of the first selected attribute is shown in Figure 2. From the top to the bottom, the importance of attributes is sorted according to the order of importance from the largest to the smallest. We can find that there are significant differences in the importance of each attribute. The 28th attribute is the most important, with a value of 0.96. The 20th attribute is the least important, which is the standard deviation of the quantitative features, with a value of only 0.05. The areas with high importance are mainly concentrated in the 21st to 24th attribute range and the 28th attribute, which are the worst values of the quantitative features, all of which are above 0.8. This shows that the worst value of the quantitative characteristics of nuclear micrograph covers a large amount of important information about data. However, the importance of the 16th, 10th, and 20th attributes is less than 0.1, which indicates that the importance of these three attributes is very low and the influence on the predictive results of breast cancer is very small, which belongs to redundancy attribute information.

The threshold value of attribute selection based on RF is set to 0.1, the attributes whose importance is lower than the threshold value are deleted, and the remaining 27 attributes are selected as the result of RF initial attribute selection. 27 attributes of the first reduction are continued to be selected by RF. We delete the redundant attributes whose importance is lower than the threshold value, calculate the importance of the remaining attribute sets and each attribute in it, and arrange them in descending order of importance.

**FIGURE 3:**
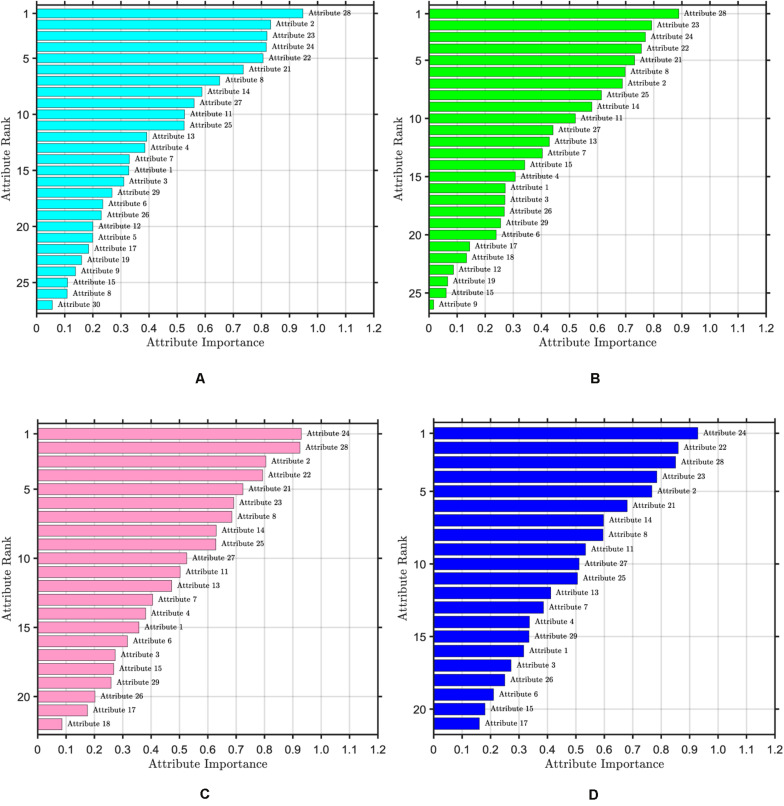
Ranking of attribute importance. The threshold value of attribute selection based on RF is set to 0.1. **(A)** Ranking of attribute importance after one iteration, including 27 attributes. **(B)** Ranking of attribute importance after two iteration, including 26 attributes. **(C)** Ranking of attribute importance after three iteration, including 22 attributes. **(D)** Ranking of attribute importance after four iteration, including 21 attributes.

The ranking of attribute importance for four iterations is shown in Figure 3. Because of the randomness of RF, it can be seen from Figures 3A,B that there are differences in the ranking of the importance of the first two attributes. The maximum value of attribute importance for both iterations is obtained at the 28th attribute. After the first iteration, only the 30th attribute is below the threshold, while the second is the 12th, 19th, 15th, and 9th attributes. As can be seen from Figures 3C,D, compared with the previous two iterations, the attribute of the maximum importance has changed, which is the 24th attribute. After the third iteration, only the importance of the 18th attribute is below the threshold, and after the fourth iteration, the importance of all attributes is greater than the threshold. We take the average attribute importance and out of bag error as the evaluation indexes of attribute selection based on RF. The larger the average attribute importance of attributes and the smaller the out of bag error, the more useful information these attributes contain, the less redundant information they have. The evaluation indexes of five iterations are presented in Table 1. From the table, we can see that with the increasing number of iterations, redundant attributes are gradually eliminated, and the corresponding evaluation indicators are also changing. When the number of iterations is 4, the average attribute importance reaches a maximum of 0.5214, the out of bag error reaches a minimum of 0.0318, and the number of attributes selected by RF is 21. In the fifth iteration, the importance of each attribute is still greater than the threshold, the number of attributes selected by RF remains unchanged, and each attribute retains the relatively important and effective information of breast cancer data. Finally, 21 attributes selected by four iterations are the result of the attribute selection of the RF algorithm.

**TABLE 1:** Evaluation indexes of five iterations.

**Iterative number**	**Attributes**	**Average attribute importance**	**Out of bag error**
1	27	0.4315	0.0335
2	26	0.4381	0.0320
3	22	0.4792	0.0337
4	21	0.5214	0.0318
5	21	0.4987	0.0322

### Feature Extraction Based on Principal Component Analysis

After RF selection, the number of attributes is reduced by 9 compared with the original data, and there is a lot of redundant information in these 9 attributes. In order to achieve the requirement of accurate prediction of breast cancer, PCA needs to be used to further simplify the data attributes. When PCA is used to extract features, to prevent PCA from over capturing some features with large values, which results in the loss of a large amount of information and the impact of features with large values on the results, we will standardize each feature first, so that their sizes are within the same range. PCA is employed to extract the 21 attributes of breast cancer data after attribute selection, and the cumulative contribution rate is 95%.

The 160 samples of each group are selected, and a total of 320 samples of breast cancer data are used as the training set. The remaining 40 samples of each group are selected, and a total of 80 samples of breast cancer data are used as the test set. [0, 1], [−1, 1], and *Z*-score normalization methods are used to normalize the breast cancer data after feature selection. The training set is used to establish the predictive model of breast cancer based on ELM, and the test set is used to test the prediction ability of the model. Under different standardized methods, we input the data of feature extraction into the predictive model of ELM, and compare their prediction accuracy of the training set and test set, then select the best normalization method.

The predictive results of different normalization methods are shown in Table 2. It can be seen that the main component scores of [0, 1] and [−1, 1] normalization methods are only two, and the accuracy of the training set is relatively low. The prediction accuracy of the *Z*-score of the training set and test set is significantly higher than the other two methods, which fully shows that the proper selection of the data standardization method plays a key role. *Z*-score is selected as the normalization method of data.

**TABLE 2:** Predictive results of different normalization methods.

**Normalization method**	**Principal components**	**Predictive accuracy/%**	
		**Training set**	**Test set**
	2	90.94 (291/320)	96.25 (77/80)
[−1,1]	2	90.63(290/320)	95 (76/80)
*Z*-score	7	99.06 (317/320)	98.75 (79/80)

**FIGURE 4:**
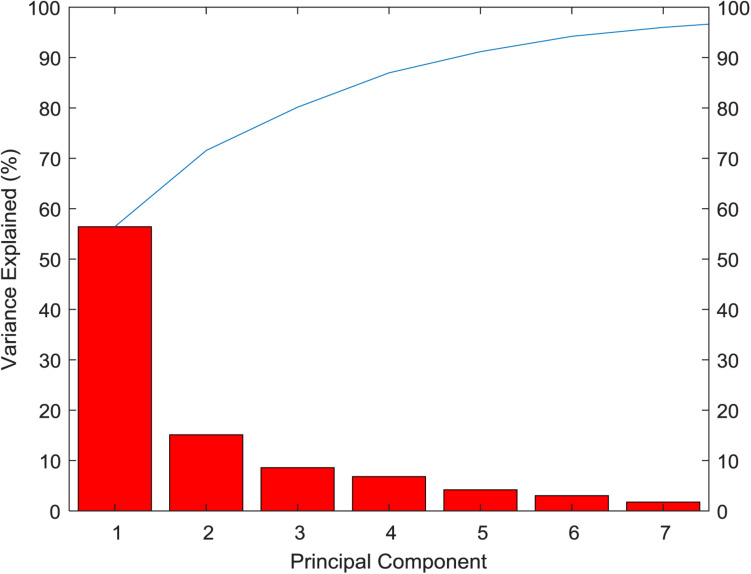
Variance contribution rate of the principal components.

From the variance contribution rate of the principal components in Figure 4, we can see that the first principal component bears 56.43% of the difference. The variance contribution rate of the first principal component is the largest, and the variance contribution rate of the other principal components is decreasing in turn, then seven principal components can be obtained. The cumulative contribution rate of principal components is shown in Table 3. The cumulative contribution rate of the first seven principal components is 95.99%, which achieves the goal of 95%. Therefore, the first seven principal components are selected as the feature of PCA extraction. Finally, the dimension of breast cancer data is reduced to 7 dimensions, which is more conducive to the subsequent recognition and prediction of breast cancer.

**TABLE 3:** Cumulative contribution of principal components.

**Principal component**	**PCA1**	**PCA2**	**PCA3**	**PCA4**	**PCA5**	**PCA6**	**PCA7**
Cumulative contribution rate/%	56.43	71.56	80.15	86.97	91.17	94.22	95.99

### Predictive Models for Breast Cancer

The prediction performance of the ELM model is affected by the type of activation function. By comparing and analyzing the predictive results of breast cancer under three different activation functions of sin, hardlim and sigmoid, the activation function with the best prediction effect was selected. Seven feature data are used to establish the predictive model of ELM under different activation functions, and the predictive results are shown in Table 4. When the Sigmoid function is used as the ELM activation function, both the training set and the test set have higher prediction accuracy.

**TABLE 4:** Predictive results of different activation functions.

**Activation function**	**Time/s**	**Predictive accuracy/%**		**Hidden layer neurons**
	**Training samples**	**Training set**	**Test set**	
Sin	0.0067	97.81 (313/320)	95 (76/80)	104
Hardlim	0.0029	98.13 (314/320)	98.75 (79/80)	53
Sigmoid	0.0022	99.06 (317/320)	98.75 (79/80)	27

In the predictive model of ELM, the number of input layer neurons, hidden layer neurons, and output layer neurons and network structure should be determined. The number of extracted features is 7, so the number of input layer neurons is 7. Because two types of breast tumors are predicted, the number of output neurons is 2. The number of hidden layer neurons is the key parameter that affects the prediction ability and generalization performance of ELM. The initial number of neurons in the hidden layer is set to 1. It is necessary to analyze the prediction of breast cancer by the ELM model corresponding to the number of different hidden layers. In order to reduce the training time of the model, the number of hidden layer neurons is set within 200.

**FIGURE 5:**
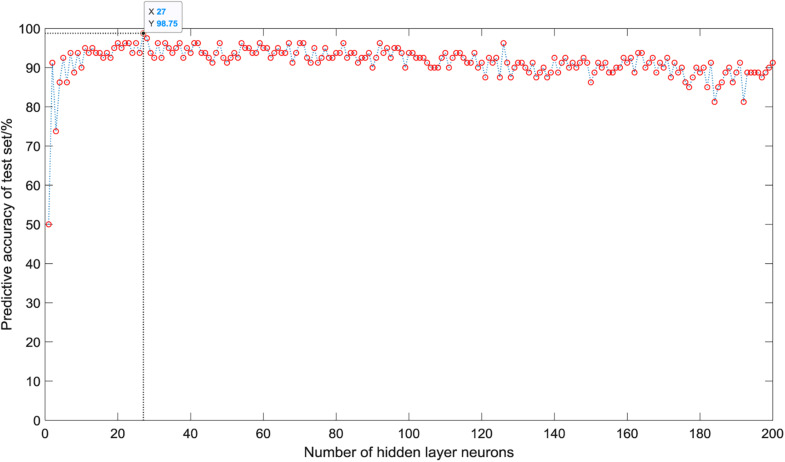
Predictive accuracy of different hidden layer neurons.

**FIGURE 6:**
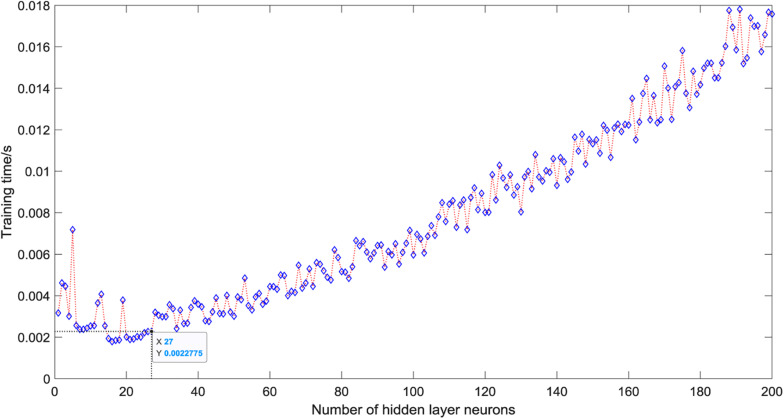
Training time of different hidden layer neurons.

As shown in Figure 5, when there is only one neuron in the hidden layer, the prediction accuracy of the test set is only 50%. When the number of hidden layer neurons is 2, the prediction accuracy increases to 91.25%. The number of neurons increases from 3 to 5, the prediction accuracy gradually increases to a higher value of 92.5%, and then began to fluctuate in the range of 81∼99%. The overall trend is relatively stable, and the average accuracy is about 92%. However, it is not that the more the number of hidden layer neurons, the better the prediction effect of the model. After the number of hidden layer neurons reaches 120, the accuracy of the test set fluctuates greatly, ranging from 81 to 96%, and the average accuracy is about 90%. When the number of hidden layer neurons is 27, the ELM model has the best prediction effect on the test set, and the prediction accuracy reaches 98.75%. Figure 6 shows the relationship between the number of hidden layer neurons and the training time. We can find that with the increase in the number of hidden layer neurons, the overall training time is on the rise. Compared with Figure 5, when the prediction accuracy reaches the maximum, the number of hidden layer neurons is 27, and the training time is only 0.0022 s.

In order to prove the reliability of attribute selection and feature extraction algorithm for breast cancer data modeling, the predictive results of the original data, the data after attribute selection, and the data after feature extraction are compared and analyzed, and the results are shown in Table 5. It can be seen that the accuracy of the training set and test set after dimension reduction is higher than that of original data modeling, which shows that attribute selection and feature extraction methods improve the predictive learning ability of model training and test samples. The number of features obtained by single RF and PCA dimensionality reduction methods is less than that of the original data, and the number of features is reduced to 70 and 33% of the original data, respectively. RF combined with PCA (RF-PCA) process the original data to get the least number of features and the number of features is only 23% of the original data. The accuracy of the training set and test set is not only higher than that of original data modeling but also higher than that of single RF and single PCA modeling. Because the classifiers used are ELM, so there is little difference in training time, only about 0.002 s, and the number of hidden layer neurons corresponding to the optimal accuracy is different.

**TABLE 5:** Predictive results of different dimensionality reduction methods.

**Dimension reduction method**	**Features**	**Predictive accuracy/%**		**Time/s**	**Hidden layer neurons**
		**Training set**	**Test set**	**Training samples**	
ELM	30	95.31 (305/320)	95 (76/80)	0.0020	14
RF + ELM	21	97.5 (312/320)	96.25 (77/80)	0.0023	24
PCA + ELM	10	97.19 (311/320)	97.5 (78/80)	0.0028	13
RF-PCA + ELM	7	99.06 (317/320)	98.75 (79/80)	0.0022	27

In order to verify the superiority of the predictive model based on breast cancer data after RF-PCA dimensionality reduction, we also compared and analyzed the prediction performance of several different modeling methods based on the data after dimension reduction, such as a PNN, SVM, BP neural network, and DT. The optimal parameter *spread* of PNN is set to 0.87. The radial basis function (RBF) is used as a kernel function of SVM. SVM uses a fivefold cross-validation method to find the best penalty coefficient *C* and kernel function parameter *g* in the range of [2^−10^, 2^10^], at which point, *C* = 2.2974, *g* = 0.0625. BP adopts the same network structure as ELM, in which the number of hidden layer neurons is 27, the learning step of BP is set to 0.3, the minimum mean square error is set to 10^–8^, and the minimum gradient is set to 10^–20^.

The predictive results of different modeling methods are shown in Table 6. According to the accuracy (Acc), precision (Pr), sensitivity (Se), specificity (Sp), F1-score (F1) and MCC, we find that although the accuracy and other evaluation indexes of the BP training set is as high as 100% and higher than that of other models. The accuracy of the test set are the lowest and other evaluation indexes are relatively low, which indicates that BP based on gradient descent method has slight over-fitting. The training time of BP is 9.6259 s, and the prediction speed is obviously slower than other methods. The accuracy of the test set of PNN, SVM, and DT is 95%, and their MCC are all 0.9, which shows that they have similar prediction performance, and the difference is mainly reflected in the evaluation index of the training set and training time. In the comparison of these three methods, the training set of PNN has the highest prediction performance and the training speed of PNN is the fastest. Finally, by comprehensively comparing the evaluation indexes of training time, Acc, Pr, Se, Sp, F1, and MCC, we can clearly see that the training time of ELM is much faster than other models, and the evaluation index of predictive performance is better than other models, which fully verifies the superiority of RF-PCA combined with ELM, and meets the requirements of real-time breast cancer auxiliary diagnosis.

**TABLE 6:** Predictive results of different modeling methods.

**Modeling method**	**Time/s**	**Training set**						**Test set**					
	**Training**	**Acc**	**Pr**	**Se**	**Sp**	**F1**	**MCC**	**Acc**	**Pr**	**Se**	**Sp**	**F1**	**MCC**
PNN	0.0339	99.69%	99.38%	100%	99.38%	99.69%	0.99	95%	95%	95%	95%	95%	0.9
SVM	1.4601	99.06%	98.16%	100%	98.13%	99.07%	0.98	95%	97.37%	92.5%	97.5%	94.87%	0.9
BP	9.6259	100%	100%	100%	100%	100%	1	93.75%	92.68%	95%	92.5%	93.83%	0.88
DT	0.1669	98.13%	98.13%	98.13%	98.13%	98.13%	0.96	95%	95%	95%	95%	95%	0.9
ELM	0.0022	99.06%	98.16%	100%	98.13%	99.07%	0.98	98.75%	97.56%	100%	97.5%	98.76%	0.98

The same algorithm can be applied to different data sets to ensure the reliability of the algorithm. If the algorithm proposed in this article can achieve good prediction results for different data sets, it can show that the algorithm has strong adaptability and generalization performance. The generalization performance of the algorithm is verified by the data (Jossinet, 1996) in UCI database. The data was obtained by jossinet’s team using electrical impedance tomography to measure the impedance of 106 pathological breast tissue from 64 women. The sample were divided into pathological tissue and normal tissue, according to the pathology and morphology of the breast. Among them, pathological tissue includes mastopathy: benignant and non-inflammatory disease of the breast (MA), fibro-adenoma (FA) and carcinoma (CA), while normal tissue includes mammary gland (MG), connective tissue (CT) and adipose subcutaneous fatty tissue (AT). A total of 80 samples were randomly divided into training sets and the remaining 26 samples were used as test sets. Firstly, RF is used for attribute selection, and then PCA is used for feature extraction. Finally, the dimension of data is reduced from 9 to 4 dimensions. The reduced dimension data of RF-PCA is fed into ELM and a predictive model is established.

We also compare common methods of classifiers used in the literature about breast cancer recognition for the new data. The reduced dimension data is fed into other classifiers and a predictive model is established. When the number of neurons in the hidden layer was 97, the ELM model has a best prediction performance. The optimal parameter *spread*of PNN is set to 0.68. The optimal *C* of SVM is 42.2243 and *g* is 9.1896. Table 7 is the comparison between the predictive results of the ELM model of the data after dimensionality reduction by RF-PCA and the raw data. It can be seen that the prediction performance of the training set and the test set of the ELM model established by the dimensionality reduction method of RF-PCA is higher than that of the ELM model established by the raw data. All the samples of the training set are predicted correctly, and only one sample of the test set is predicted incorrectly. The number of features of the data is almost reduced to half of the raw data, and the training time is only about 0.0011 s. In the training set, we find that the prediction performance of PNN is the worst. BP has the same prediction performance as ELM, but the training time is the longest. In the test set, SVM has a similar prediction performance as ELM, and a faster training speed. BP has the worst prediction performance. Comparing all kinds of evaluation indexes of the model, it can be seen that ELM and SVM have a good prediction effect and the fast training time in the electrical impedance data, but the performance of the model established by the method of BP is poor.

**TABLE 7:** Predictive results of dimensionality reduction by RF-PCA.

**Modeling method**	**Time/s**	**Training set**						**Test set**					
	**Training**	**Acc**	**Pr**	**Se**	**Sp**	**F1**	**MCC**	**Acc**	**Pr**	**Se**	**Sp**	**F1**	**MCC**
Raw + ELM	0.0096	95%	85.71%	100%	85.71%	92.31%	0.86	92.31%	92.86%	97.5%	92.5%	95.12%	0.9
ELM	0.0011	100%	100%	100%	100%	100%	1	96.15%	92.31%	100%	92.86%	96%	0.93
PNN	0.0314	91.25%	94.59%	87.5%	95%	90.91%	0.83	88.46%	80%	100%	78.57%	88.89%	0.79
SVM	0.1592	97.5%	95.24%	100%	95%	97.56%	0.95	96.15%	100%	91.67%	100%	95.65%	0.93
BP	1.3080	100%	100%	100%	100%	100%	1	84.62%	78.57%	91.67%	78.57%	84.62	0.7
DT	0.0551	96.25%	93.02%	100%	92.5%	96.39%	0.93	92.31%	91.67%	91.67%	92.86%	91.67%	0.85

All of these shows that the method proposed in this article can still achieve a better prediction performance and faster speed when applied to the new dataset to predict new samples. To a certain extent, the proposed method can exclude the possibility of overfitting of the models.

## Conclusion

In this article, we put forward a new solution based on attribute selection and feature extraction for rapid diagnosis of breast cancer, which is called RF-PCA. Firstly, we used the attribute selection based on RF of algorithm to select the useful attributes of quantitative feature data of breast tumor cell images and then used the feature extraction algorithm based on PCA to reduce the dimension of data after attribute selection. Finally, the ELM model was established to test the prediction effect of breast cancer. In order to verify the reliability of this algorithm, we compared the prediction accuracy of ELM model after using RF or PCA alone. To verify the superiority of this algorithm, we also compared the prediction performance of different models and used the impedance data of the breast tissue to verify the adaptability of the algorithm.

The results show that (1) The feature selection based on RF or feature extraction based on PCA of a method can not only reduce the complexity of the training model but also improve the prediction accuracy of the model to a certain extent; (2) Combining feature selection with feature selection, we use the advantages of the two methods to reduce the dimension of data. Compared with the single dimension reduction method, it can reflect the effective information of the original data with fewer features, make the model simple, and improve the efficiency and reliability of modeling; (3) ELM model has high prediction accuracy and short training time, which effectively avoids over-fitting and has a certain generalization ability; (4) RF-PCA combined with ELM model can significantly reduce the training time of the network, and more adapt to the requirements of a rapid and accurate breast cancer aided diagnosis.

Despite the achievement of some research results, there are some limitations in this study. When the proposed algorithm in this article is used in breast cancer diagnosis, the training time is reduced and the prediction accuracy is better. However, these advantages mainly focus on the fast prediction speed and does not reach the optimal accuracy of all samples. Therefore, in future work, it will be necessary to study some optimization algorithms to improve the performance of the model and achieve the highest prediction accuracy on the basis of ensuring faster prediction speed.

## Data Availability Statement

Publicly available datasets were analyzed in this study. This data can be found here: http://archive.ics.uci.edu/ml/datasets/Breast+Cancer+Wisconsin+%28Diagnostic%29, https://archive.ics.uci.edu/ml/datasets/Breast+Tissue. We have uploaded the code by using Matlab to GitHub. With this URL (https://github.com/bkfly/test.git), you can easily download the code of this article. In addition, you can visit https://help.github.com/en#dotcom for more instructions on the use of GitHub.

## Author Contributions

KB conceived the study. MZ developed the method and supervised the study. KB and WL implemented the algorithms. KB and FH analyzed the data. KB wrote the manuscript. All authors read and approved the final version of the manuscript.

## Conflict of Interest

The authors declare that the research was conducted in the absence of any commercial or financial relationships that could be construed as a potential conflict of interest.
