# Supplementary material for: RF-PCA: A New Solution for Rapid Identification of Breast Cancer Categorical Data Based on Attribute Selection and Feature Extraction
Source: Front Genet. 2020 Sep 9;11:566057. doi: 10.3389/fgene.2020.566057 (PMC7510777; doi:10.3389/fgene.2020.566057)
Supplement: Supplementary file 1 [file Data_Sheet_1.PDF]

Supplementary Material

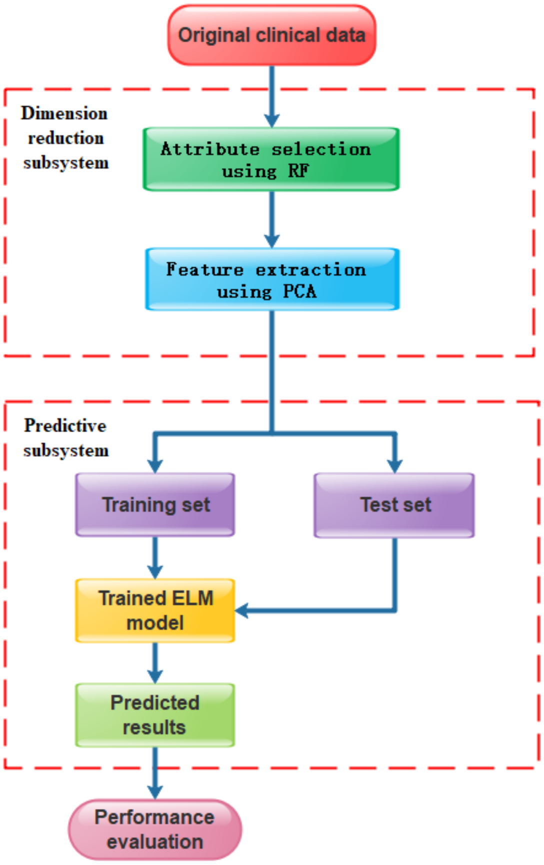

Supplementary Figure 1. A workflow about rapid identification of breast cancer categorical data

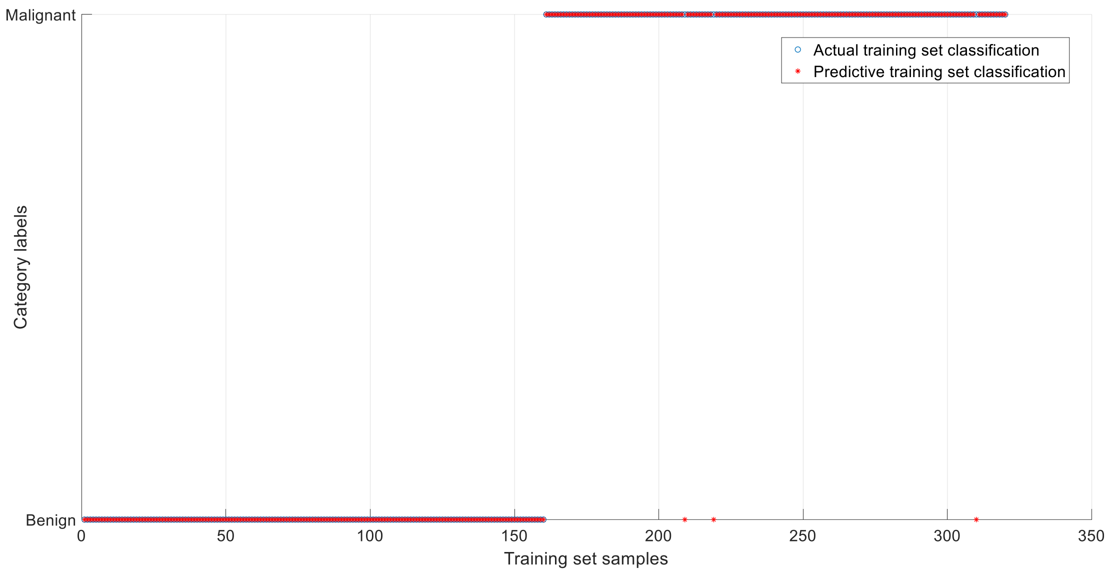

Supplementary Figure 2. Actual classification and predictive classification of the training set

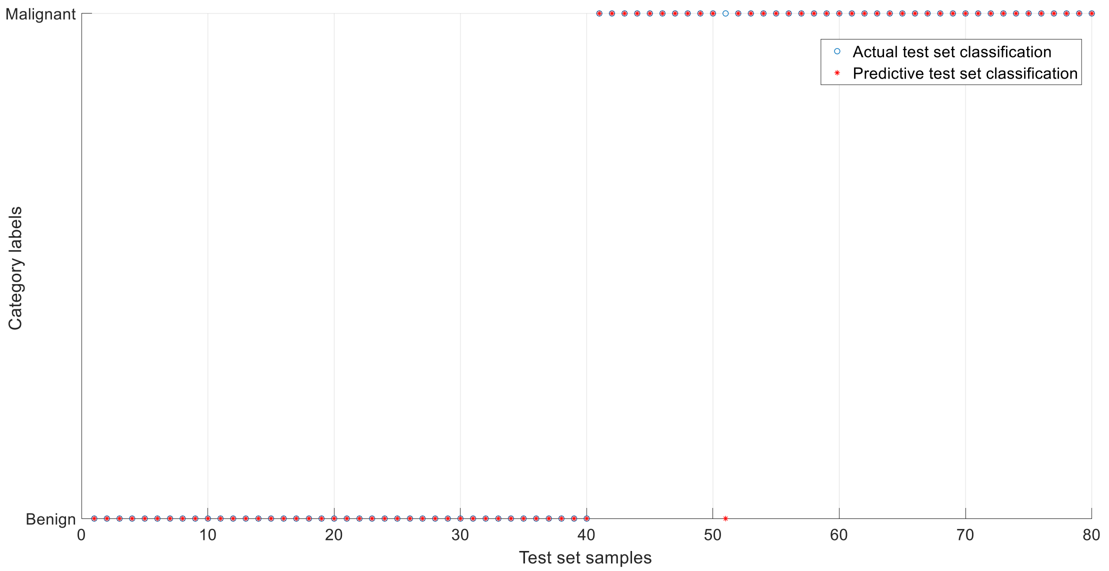

Supplementary Figure 3. Actual classification and predictive classification of the test set

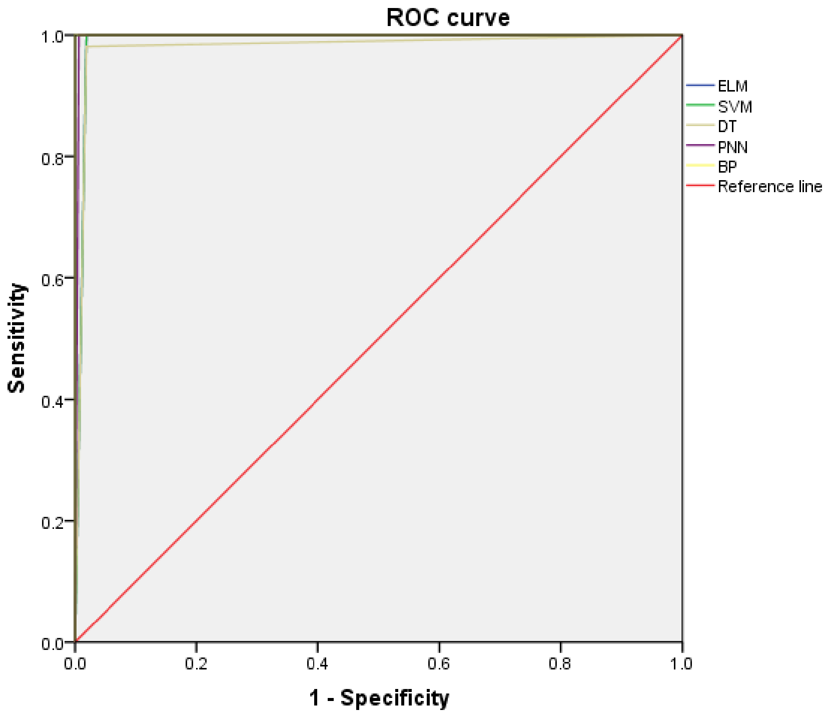

Supplementary Figure 4. Comparison of ROC curves in training sets

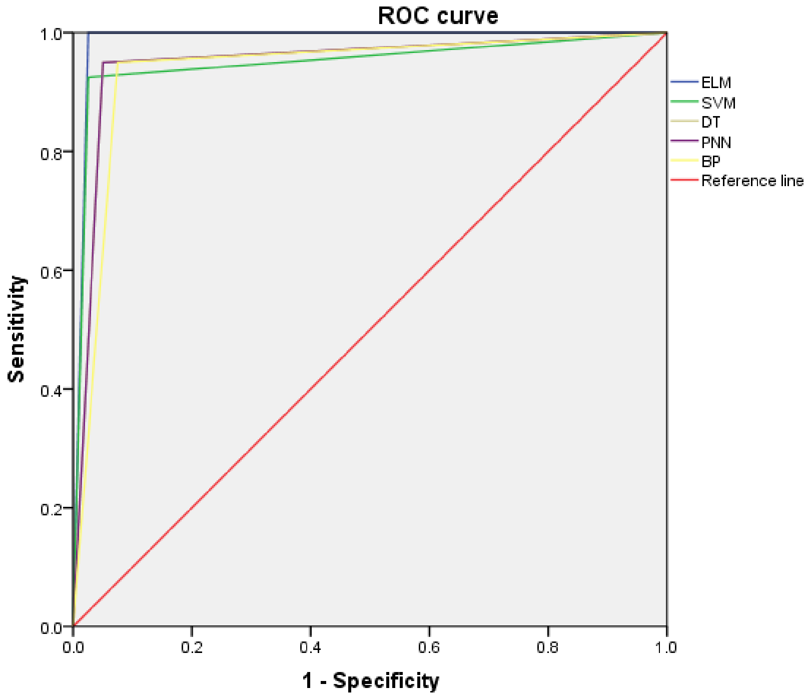

Supplementary Figure 5. Comparison of ROC curves in test sets

Supplementary Table 1. The results of quantitative features of data

Attributes	Quantitative features (range of values)		
	Mean	Standard error	Maximum
Radius	6.98-28.11	0.112-2.873	7.93-36.04
Texture	9.71-39.28	0.36-4.89	12.02-49.54
Perimeter	43.79-188.5	0.76-21.98	50.41-251.2
Area	143.5-2501	6.8-542.2	185.2-4254
Smoothness	0.053-0.163	0.002-0.031	0.071-0.223
Compactness	0.019-0.345	0.002-0.135	0.027-1.058
Concavity	0-0.427	0-0.396	0-1.252
Concave points	0-0.201	0-0.053	0-0.291
Symmetry	0.106-0.304	0.008-0.079	0.157-0.664
Fractal dimension	0.05-0.097	0.001-0.030	0.055-0.208

Supplementary Table 2. Confusion matrix of training set and test set

Actual class	Predicted class			
	Training set		Test set	
	Benign	Malignant	Benign	Malignant
Benign	160(TP)	0(FN)	40(TP)	0(FN)
Malignant	3(FP)	157(TN)	1(FP)	39(TN)
